# Substrate-independent immunomodulatory characteristics of mesenchymal stem cells in three-dimensional culture

**DOI:** 10.1371/journal.pone.0206811

**Published:** 2018-11-08

**Authors:** Jing Li, Tong Chen, Xiahe Huang, Yunshan Zhao, Bin Wang, Yanyun Yin, Yi Cui, Yannan Zhao, Ruiping Zhang, Xiujie Wang, Yingchun Wang, Jianwu Dai

**Affiliations:** 1 Laboratory of Translational Medicine, Chinese PLA General Hospital, Beijing, China; 2 State Key Laboratory of Molecular Developmental Biology, Institute of Genetics and Developmental Biology, Chinese Academy of Sciences, Beijing, China; 3 University of Chinese Academy of Sciences, Beijing, China; 4 The State Key Laboratory of Plant Genomics, Institute of Genetics and Developmental Biology, Chinese Academy of Sciences, Beijing, China; 5 State Key Laboratory of Molecular Developmental Biology, Institute of Genetics and Developmental Biology, Chinese Academy of Sciences, Beijing, China; 6 Institute of General Surgery, Chinese PLA General Hospital, Beijing, China; 7 Department of Radiology, First Clinical Medical School of Shanxi Medical University, Taiyuan, Shanxi, China; The University of Adelaide, AUSTRALIA

## Abstract

Mesenchymal stem cells (MSCs) play important roles in tissue regeneration, and multi-lineage differentiation and immunomodulation are two major characteristics of MSCs that are utilized in stem cell therapy. MSCs *in vivo* have a markedly different three-dimensional (3D) niche compared to the traditional two-dimensional (2D) culture *in vitro*. A 3D scaffold is predicted to provide an artificial 3D environment similar to the *in vivo* environment. Significant changes in MSC differentiation are shown to be occurred when under 3D culture. However, the immunomodulatory characteristics of MSCs under 3D culture remain unknown. In this study, 3D culture systems were constructed using different substrates to evaluate the common immunomodulatory characteristics of MSCs. Compared to the MSCs under 2D culture, the MSCs under 3D culture, which had higher stemness and maintained cell phenotype, showed altered immunophenotypic pattern. Gene expression profile analysis at mRNA and protein level detected by gene chip and protein chip, respectively, further revealed the difference between 3D cultured MSCs and 2D cultured MSCs, which was mainly concentrated in the immunoregulation related aspects. Moreover, the immunoregulatory role of 3D culture was confirmed by our immunosuppressive experiments. These findings demonstrated that the immunomodulatory capacities of MSCs were enhanced by the 3D geometry of substrates.

## Introduction

Mesenchymal stem cells (MSCs) are an adult multipotent stem cell population residing in various tissues including bone marrow, adipose tissue, umbilical cord, and placenta. As a type of adult stem cell, MSCs have two distinct characteristics that distinguish from other adult cells types. First, they have “stemness”, although it is not completely defined, which is demonstrated by their potential for self-renewal and tri-lineage differentiation into osteoblasts, chondrocytes, and adipocytes [1–4]. Second, MSCs have immunomodulatary characteristics under specific condition. Studies have revealed that, although cell replacement has an important role in MSC therapy for some diseases, the ultimate therapeutic effect is predominantly an outcome of immunomodulatary capacity derived from MSCs, which react with immune system [4, 5]. This immunomodulatary capacity in MSC therapy primarily presents as immunosuppression in animal models and human studies [4, 6]. Based on these two major characteristics, MSCs are potentially useful for tissue repair and regeneration in stem cell therapy.

The majority of MSC characteristics were identified based on 2D culture system that is a convenient platform for MSC study and is easy to expand to produce large quantities for proposed clinical applications [7–9]. However, the niche in which MSCs reside *in vivo* is a distinct 3D environment, and different from that in traditional 2D culture *in vitro* [10]. The in vivo 3D environment could affect cell surface topography even cell morphology, which could further influence the characteristics of MSCs by different cell-cell and cell-extracellular matrix contacts. The changes in cell topography could directly or indirectly influence cell adhesion, migration, self-renewal and differentiation of MSCs [11–13], but whether immunomodulatary characteristics of MSCs are also affected by geometry remains unknown.

A 3D culture system provides 3D environment for MSC culture, which mimics an *in vivo* niche. Numerous substrates have been used for constructing 3D culture system of MSCs. However, there is no uniform conclusion on the characteristics of MSCs under different 3D scaffolds [14–16]. The different substrates might provide different 3D geometry for MSCs and activate/suppress different signaling pathway, leading to distinct regulatory functions of 3D cultured MSCs [17–19]. Therefore, it is challenging to find out the common changes of MSC characteristics regardless of materials themselves.

In this study, we developed three types of 3D culture systems using three different substrates, evaluated several immunological characteristics, and performed global genome and proteome analyses to determine the common changes of MSC characteristics, particularly on immunomodulatory characteristics.

## Materials and methods

### Scaffolds

Three types of scaffolds were constructed using collagen substrate, chitosan substrate, and PLGA substrate according to previous methods [20].

### Scanning electron microscopy analysis

The morphology of the MSCs seeded on three 3D substrates was determined using scanning electron microscopy (SEM, S-3000N; Hitachi, Tokyo, Japan). First, the samples were washed with cold DPBS three times and fixed with cold 2% glutaraldehyde solution for 12 h at 4°C. Then, the samples were dehydrated in a series of ethanol (50%, 75%, 85%, 95%, 100% and 100%). The samples were critical point dried and sputter coated with gold platinum prior to SEM imaging.

### Cell culture

The MSCs were obtained from human umbilical cord tissue. The study was approved by the Ethics Committee at Institute of Genetics and development, Chinese Academy of Sciences. The fresh umbilical cords were collected at the hospital after obtaining written informed consent from each donor participating in this study. The blood vessels were removed by washing with PBS. The clean tissue was sliced into small pieces and digested with 0.1% collagenase P in Dulbecco’s modified Eagle’s medium/Ham’s F-12 medium (DF-12; Gibco, Grand Island, NY) for 3 h at 37°C. The cells were harvested from pellets after centrifugation, plated in 75-cm^2^ culture flashes at a density of 2×10^6^ cells in 12 mL of regular growth medium and incubated at 37°C with 5% CO_2_. The regular growth medium contained 96% DF-12, 2% fetal bovine serum (FBS; Gibco), 10 ng/mL epidermal growth factor, 10 ng/mL platelet derived growth factor BB (PDGF-BB) (all purchased from Peprotech), 100 U/mL penicillin, and 100 μg/mL streptomycin (Gibco).

For 2D MSCs culture, the cells were cultured according to a previous protocol. The medium was changed every three days. After seven days of culture, the cells were trypsinized with 0.5% trypsin (Sigma) and 0.1% EDTA solution at 37°C for 3–5 min. After trypsinization, an equal amount of FBS was added and the pieces were pipetted up and down several times with 10 ml PBS. The cells were collected by centrifugation at 1200 rpm for 6 min at room temperature and resuspended in fresh MSC medium. The cells were then plated on cell culture plates.

For 3D MSC culture, the substrates were immersed in MSC medium to remove the gas. After trypsinization, the MSCs cultured on plates were collected by centrifugation at 1200 rpm for 6 min at room temperature and resuspended in fresh MSC medium. The cell suspension was plated onto cell substrates with 3×10^4^ cells on a collagen substrate and 6×10^4^ cells on a chitosan or PLGA substrate. After 4–6 h, MSC medium was added to cover the substrates. The substrates were incubated overnight and then transferred to shakers at 60 rpm. The medium was changed every three days.

### Cell proliferation analysis

The proliferation rates of the cells cultured in 3D scaffolds were assayed using a hemocytometer. For cell trypsinization, 0.5% trypsin was added and incubated at 37°C for 10 min. Trypsinization was terminated with an equal amount of FBS. The cell number was determined using a hemocytometer at the indicated time points. A total of 3×10^4^ cells or 6×10^4^ cells were seeded on one substrate on day 0. The cells were detached from the substrates and the cells numbers were counted on day 1, which represented the cells attached to the substrates. The fold increase of the cell numbers was calculated by dividing by the cell numbers on day 1.

### Cell cycle analysis

For each sample, approximately 1×10^6^ cells were collected and fixed with 300 μL of PBS plus 700 μL of 70% ethanol. After fixing at 4°C overnight, the cells were resuspended with 500 μl of PBS containing 50 μg/ml PI and 100 μg/ml RNase A. The cells were incubated at 37°C for 30 min and then analyzed on a FACS (Attune, Thermo Fisher) equipped with Attune software.

### Cell differentiation induction

For each type of differentiation culture, the cells were seeded at 2–3×10^4^ cells/cm^2^ or at 4–5×10^4^ cells/slice. For adipogenic differentiation, the MSCs were induced in adipogenic differentiation medium (DMEM supplemented with 10% FBS, 1 mM dexamethasone, 0.5 mM 3-isobutyl-1-methylxanthine, and 0.1 mM ascorbic acid) for 14 days. For osteogenic differentiation, the MSCs were induced in osteogenic medium (DMEM supplemented with 0.1 mM dexamethasone, 10 mM beta-glycerophosphate, 0.05 mM ascorbic acid, and 10% FBS) for 21 days.

### Quantitative RT-PCR

Total RNA was isolated with Trizol LS Reagent (Life Technologies). After removing the residual DNA with Dnase I (Life Technologies), equal amounts of RNA (1 mg) were added to the reverse transcriptase reaction mixture (SuperScript III First-Strand Synthesis System, Life Technologies) with oligo-dT primers (Life Technologies). Quantitative PCR analysis was performed in triplicate (n = 3) using the Power SYBR Green RT-PCR Kit (Life Technologies). GAPDH was used as a normalizer to determine the other gene relative transcripts (DDCt). The primer sequences are available in S1 Table.

### Immunofluorescence assays

Cultured cells were fixed for 10 min at 4°C in ice-cold methanol, washed several times in phosphate-buffered saline (PBS), permeabilized in PBS+ 0.1% Triton X-100 for 10 min on ice and then blocked for 30 min in PBS + 0.5% Tween-20 (PBST) containing 1% BSA. Primary antibodies were incubated overnight at 4°C, washed with PBS and then incubated with the secondary antibodies at room temperature for 1 h. The incubated cells were washed in PBS, and Hochest 33342 (Sigma-Aldrich) was used to visualize the nuclei.

### Flow cytometry assays

Flow cytometric analysis was performed to analyze the MSC related markers and immune related markers. A total of 1×10^5^ cells were used in the study. The cells were incubated at 4°C for 1 h with the following primary antibodies: CD34, CD44, CD73, CD90, CD80, CD86, HLA-DR, (BD Biosciences) or isotope antibodies, which served as negative controls. The cells were analyzed using flow cytometry with the Attune (Thermo Fisher) software (BD Biosciences).

### Microarray

The total RNA from each group (MSCs in 2D surface culture, MSCs in 3D collagen substrate culture, MSCs in 3D chitosan substrate culture, and MSCs in 3D PLGA substrate culture) was analyzed using gene microarrays. The gene expression profiles were assessed using the Affymetrix u 133 plus expression microarrays. Total RNA was extracted using TRIZOL Reagent (Life technologies) following the manufacturer's instructions and verified for a RIN number to determine the RNA integrity using an Agilent Bioanalyzer 2100 (Agilent technologies). The qualified total RNA was further purified with the RNeasy mini kit (QIAGEN) and RNase-Free DNase Set (QIAGEN). The total RNA was amplified, labeled and purified using the GeneChip 30IVT Express Kit (Affymetrix) following the manufacturer's instructions. Array hybridization and washing was performed using the GeneChip Hybridization, Wash and Stain Kit (Affymetrix) in a Hybridization Oven 645 (Affymetrix) and Fluidics Station 450 (Affymetrix) following the manufacturer's instructions. The slides were scanned with GeneChip Scanner 3000 (Affymetrix) and Command Console Software 3.1 (Santa Clara) using the default settings. Raw data (S1, S2, S3 and S4 Data) were normalized with the MAS 5.0 algorithm, Gene Spring Software 11.0 (Agilent technologies).

The raw microarray data have been deposited in Gene Expression Omnibus (GEO) at NCBI (https://www.ncbi.nlm.nih.gov/geo/) under the accession number GSE115947.

Total protein from the MSCs in 2D surface culture and 3D collagen substrate culture were analyzed using protein microarray. The proteins were digested using the filter-aided proteome preparation method as previously described [21]. The resulting tryptic peptides of the 2D-MSCs and 3D-MSCs were collected and labeled with iTRAQ reagents (AB Sciex, Inc., Framingham, MA) according to the manufacturer’s instructions, with slight modifications. Protein identification and quantitation were performed using the ProteinPilot 4.5 software (AB Sciex) with the Paragon database search algorithm (4.5.0.0.1654) [22].

### MLC

PBMCs from health donors were isolated with a Ficoll-Hypaque density gradient (density, 1.077 g/L; Sigma). T cells were expanded in RPMI-1640 medium supplemented with 10% FBS and cultured for 14 days in the presence of recombinant IL-2 (20 IU/ml; R&D Systems, Minneapolis, MN, USA). The medium was changed every 3 days. T cells were then collected and analyzed by FACS (Attune, Thermo Fisher). More than 70% of the cells expressed CD3.

The suppressive effects of MSCs (P4) on mixed PBMC reaction (MLR) and T cell (responded T cells, T1) proliferation stimulated by stimulated T cells (T2) were measured using the FACS analysis. MSCs were seeded in 48-well culture plates (1X10^5^ for each well) for 6 hours for adherence. Responded T cells (T1) are from a healthy donor, and the stimulatory T cells (T2) are from another unrelated healthy donor treated with mitomycin C. The ratio of responded T cells (T1), stimulated T cells (T2) and MSCs (2D or 3D culture) was 20:1:1. To ensure efficient cell-cell contact, the reactions were performed for 5 days in 0.2 ml modified RPMI-1640 medium (Gibco) supplemented with 10% FBS, and medium was changed at day 2.5. All measurements were performed using FACS analysis (Attune, Thermo Fisher).

### Statistical analysis

One-way ANOVA test was used to compare the means of more than two samples. A value of P < 0.05 was considered statistically significant.

## Results

### 3D substrate support human MSC growth

**Fig 1:**
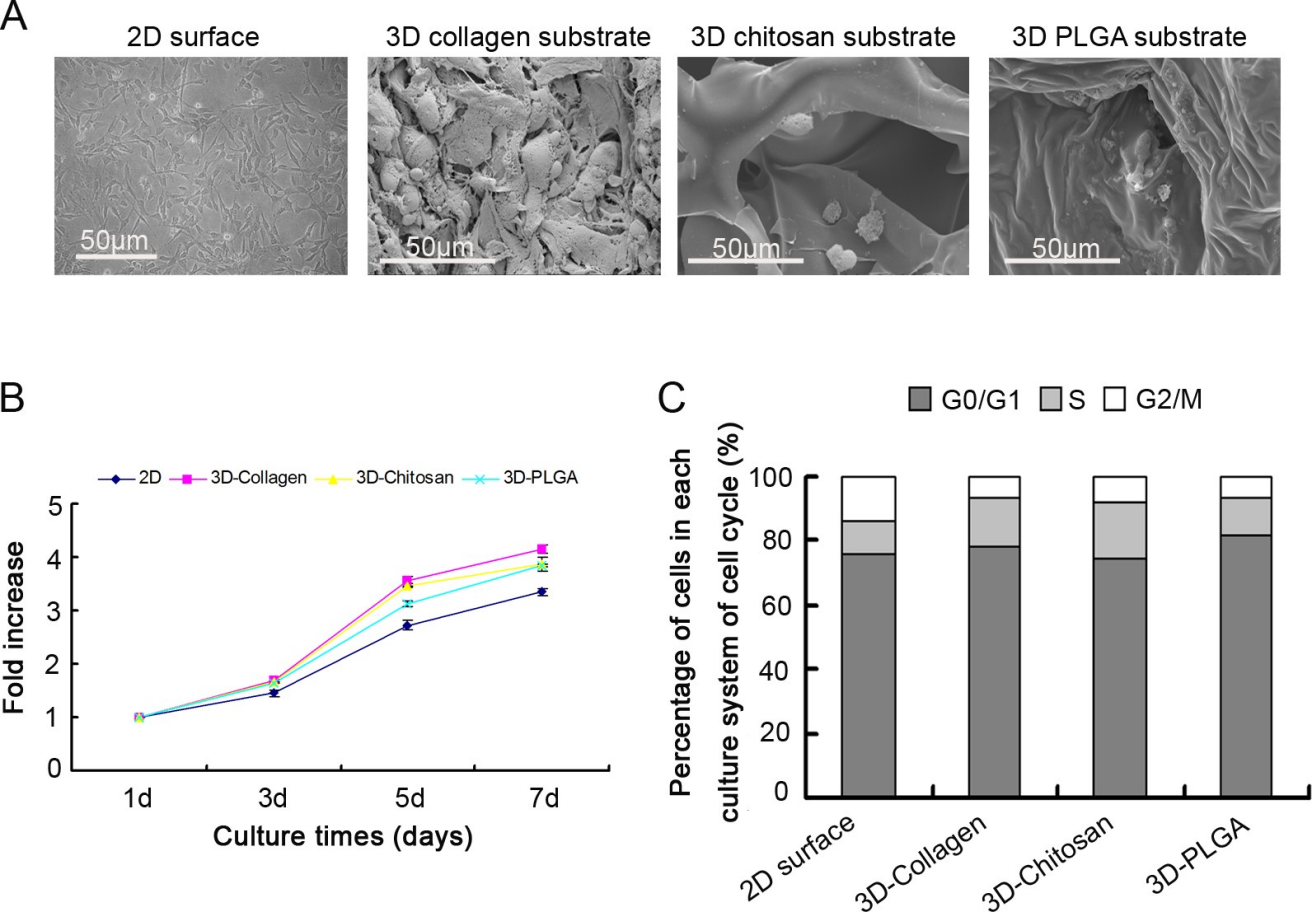
Proliferation of MSCs on different substrates. A. Light microscopy and scanning electron microscopy (SEM) images of MSCs on three different scaffolds. Scale bar = 50μm. B. Proliferation assay for MSCs cultured in different scaffolds (n = 3). C. Cell cycle assay of MSCs after 5 days culture on collagen scaffolds (3D-Collagen), chitosan substrates (3D-Chitosan) and PLGA substrates (3D-PLGA). MSCs cultured on conditional 2D surfaces served as the control.

To detect the common characteristics of MSCs under 3D culture and exclude the interference of different substrates, we first determined the growth characteristics of MSCs cultured under three different 3D culture systems constructed with collagen, chitosan and PLGA, respectively. Similar to our previous study [20], all three 3D substrates possessed a uniform porous structure. After seeded on the substrates, the MSCs attached to the surface and the internal of the substrates. The cells under the 2D surfaces showed spindle-shaped morphology. By contrast, the cells cultured under 3D substrates presented a less flat morphology (Fig 1A). There was higher proliferation rate in the 3D cultured MSCs (3D-MSCs) compared to the 2D cultured MSCs (2D-MSCs), although the difference was not statistically significant (Fig 1B). There were no significant differences in the proliferation rates for the MSCs with different 3D substrates from day 1 to day 7. Cell cycle analysis by FACS showed that all 3D-MSCs displayed a higher proportion of S phase cells compared with the 2D-MSCs (Fig 1C). These results show that these three 3D substrates support MSC adhesion and proliferation, and the proliferation rates and the cell cycles were similar for the three 3D-MSCs, indicating that the three different substrates had no interference with MSCs growth.

### 3D scaffold culture improves the stemness of MSCs

**Fig 2:**
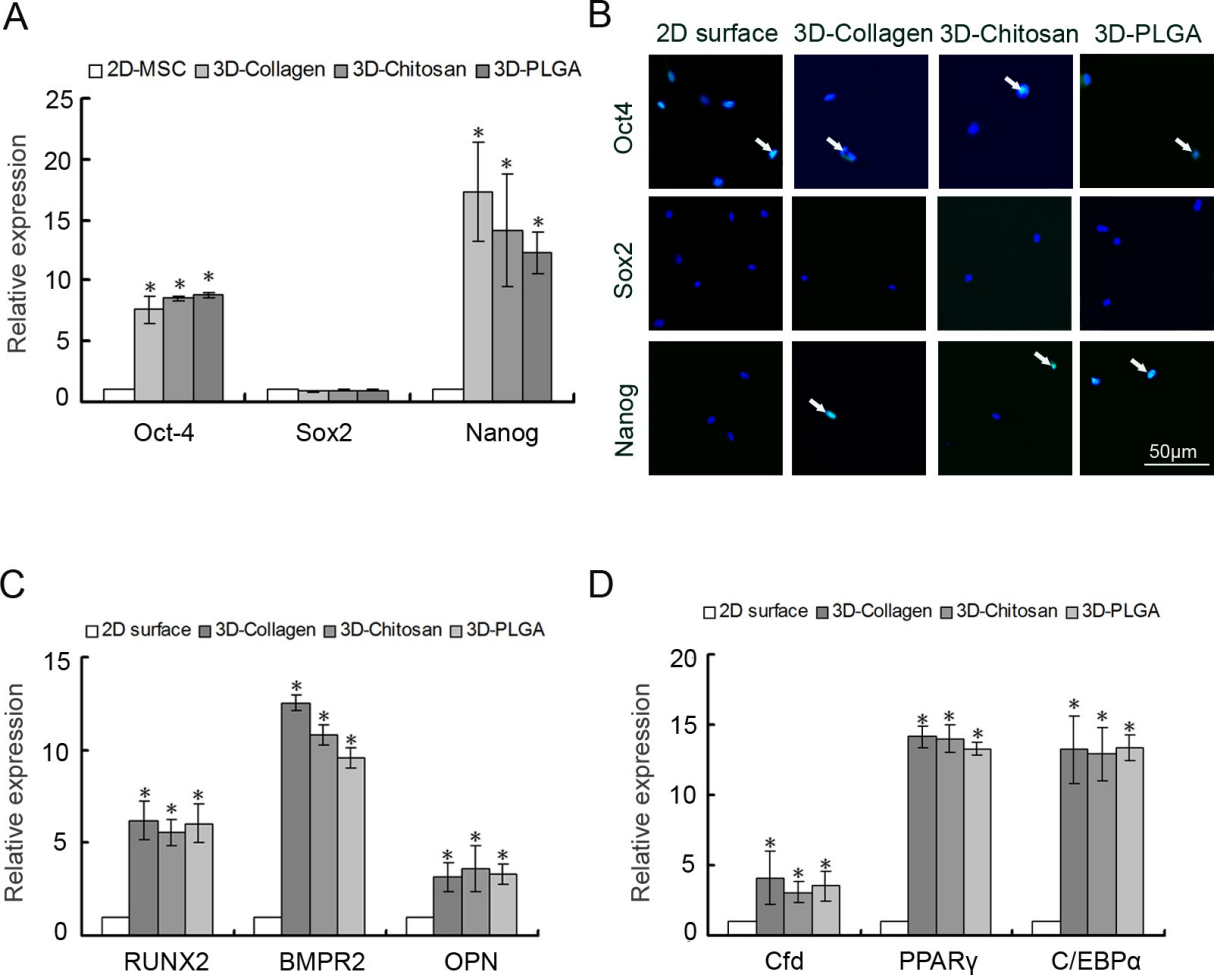
Pluripotency and differentiation of MSCs cultured on three different substrates. A. qRT-PCR for Oct4, Sox2 and Nanog of MSCs cultured in different substrates. MSCs cultured on conditional 2D surfaces served as the control. B. Immunostaining for Oct4, Sox2 and Nanog in MSCs after 5-day culture on collagen coated 2D surface (Collagen 2D) and 3D scaffolds. Scale bars = 50μm. C and D: q RT-PCR for osteogenic genes (C) and adipogenic genes (D) of MSCs cultured in different substrates after 14 days of osteogenic and adipogenic induction. MSCs cultured on conditional 2D surfaces after 14 days of osteogenic and adipogenic induction served as the control. (*) Indicates statistical significance (p<0.05) compared to the 2D surface group (n = 3).

Our previous studies have showed that 3D scaffold culture affected the stemness of mouse embryonic stem cells (ESCs) and rat MSCs [19, 20]. However, whether the stemness of human MSCs is affected by 3D scaffold culture remains unknown. Oct4, Nanog and Sox2 are core regulatory factors of the pluripotency of ESCs and are also used to mark the stemness of MSCs [23, 24]. These factors were evaluated by Realtime-PCR when MSCs were cultured for 5 days under 3D scaffold culture. The results showed that the expression of Oct4 and Nanog, not Sox2 was significantly up-regulated in 3D-MSCs compared with 2D-MSCs (Fig 2A). We then determined protein expression of core regulatory factors by immunofluorescence staining. As shown in Fig 2B, the expression of Oct4 was not significantly changed and the expression of Nanog was significantly increased after 5 days culture in the 3D-MSCs. However, there was no difference of Sox2 expression both of which was very low between the 3D-MSCs and the 2D-MSCs. We further evaluated the differentiation potential of 3D-MSCs in vitro. As shown in Fig 2C and 2D, the expression of osteogenic-related genes, such as RUNX2, BMPR2 and OPN, and adipogenic-related genes, such as Cfd, PPARγ, and C/EBPα, was significantly upregulated in 3D-MSCs compared to 2D-MSCs. The analysis revealed that human 3D-MSCs have greater osteogenic and adipogenic differentiation potential.

### 3D scaffold culture affects the immunophenotypic pattern of MSCs

**Fig 3:**
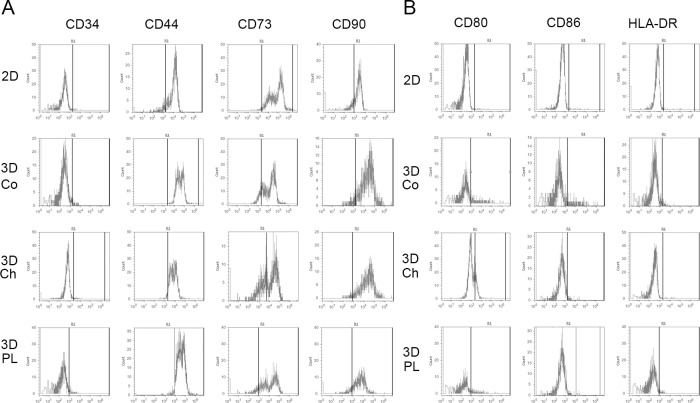
Effect of different 3D scaffolds on MSC phenotype. Mesenchymal stromal cells (MSCs) were collected after 5 days of culture in the absence or presence of different 3D scaffolds and stained with the appropriate antibodies. A. MSC-related markers are shown. B. Immunological markers are shown.

Evaluation of the stemness of 3D-MSCs identified the 3D-MSCs as stem cells. In this section, we tested the influence of 3D culture on the immunophenotypic pattern of MSCs. The selection of markers was based on the minimal criteria to define MSCs [25, 26]. As shown in Fig 3A, 3D-MSCs positively expressed CD44, CD90, and negatively expressed CD34, with no differences compared to the 2D-MSCs. However, CD73 expression was decreased in all 3D-MSCs compared to the 2D-MSCs in regular growth medium. The different surface markers indicated that the 3D-MSCs might have a different immunomodulatory status. We then analyzed the expression of molecules related to the immunogenicity of MSCs. Neither 3D-MSCs nor 2D-MSCs express CD80, CD86, and HLA-DR (Fig 3B). These results demonstrate that 3D-MSCs have the same low immunogenicity as the 2D-MSCs.

### 3D scaffold culture alters the genome expression profiles of MSCs

**Fig 4:**
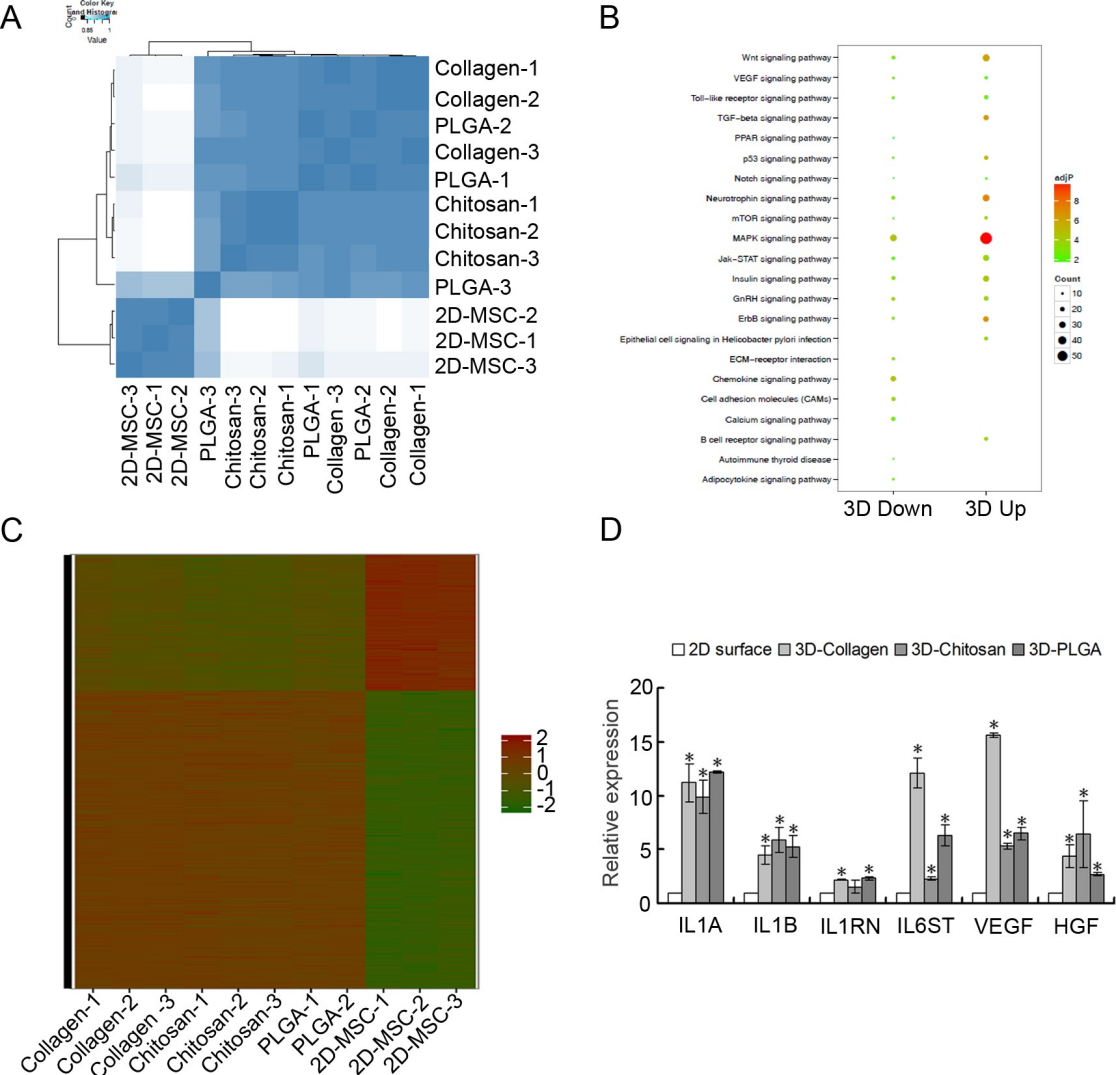
Microarray analysis of MSCs cultured on 3D scaffolds and 2D scaffolds. A. Clustering analysis of MSC whole gene expression after 5-day culture on different 3D substrates and 2D surfaces. The color from shallow to deep indicates that the correlation between samples gradually increased. B. Diagram of Kyoto Encyclopedia of Genes and Genomes (KEGG) analysis of differentially expressed genes. C. Clustering analysis with a set of immune related genes. D. qRT-PCR for immune modulatory related genes of MSCs. MSCs cultured on traditional 2D surfaces served as the control. (*) Indicates statistical significance (p<0.05) compared to the 2D surface group (n = 3).

To further understand the effect of 3D culture on MSC immunological capacities, whole genome expression profiles of 2D-MSCs and all 3D-MSCs were compared by microarray analysis. Clustering analysis of the gene expression microarray revealed that the gene expression patterns had great variation between the 3D-MSCs and 2D-MSCs, but the different 3D-MSCs showed a high degree of similarity for the gene expression patterns (Fig 4A). Compared with the 2D-MSCs, all 3D-MSCs have 3,600 up-regulated genes and 1,542 down-regulated genes. Of them, 3,328 up-regulated genes and 1,460 down-regulated genes were not different among the 3D-MSCs. A total of 354 differentially expressed genes occurred in all three 3D scaffold culture. Based on the Kyoto Encyclopedia of Genes and Genomes (KEGG) analysis, many of the differentially expressed genes were enriched in signaling pathways involved in the immunomodulatory capacities of MSCs. For example, the WNT signaling pathway, toll-like receptor signaling pathway, TGF-β signaling pathway and ECM-receptor interaction pathway were identified, which play important roles in the immunomodulatory process of MSCs (Fig 4B). This analysis indicated that the geometry could affect immunomodulatory characteristics of MSCs. Immunomodulation related genes were also selected for clustering to analyze the genotype or phenotype. As shown in Fig 4C, the samples were divided into two groups by culture geometry (dimensionality) according to immunomodulation related gene expression. The differences in the immune system processes between 2D-MSCs and all 3D-MSCs were significant, and suggest that 3D-MSCs have different immunomodulatory capacities compared to 2D-MSCs. We also examined the expression of immunomodulation-related genes of MSCs. Compared to 2D-MSCs, the expression of IL1A, IL1B, IL1RN, IL6ST, HGF and EGF was significantly up-regulated in 3D-MSCs, which is consistent with the gene microarray analysis (Fig 4D).

### 3D scaffold culture alters the protein expression profiles of MSCs

**Fig 5:**
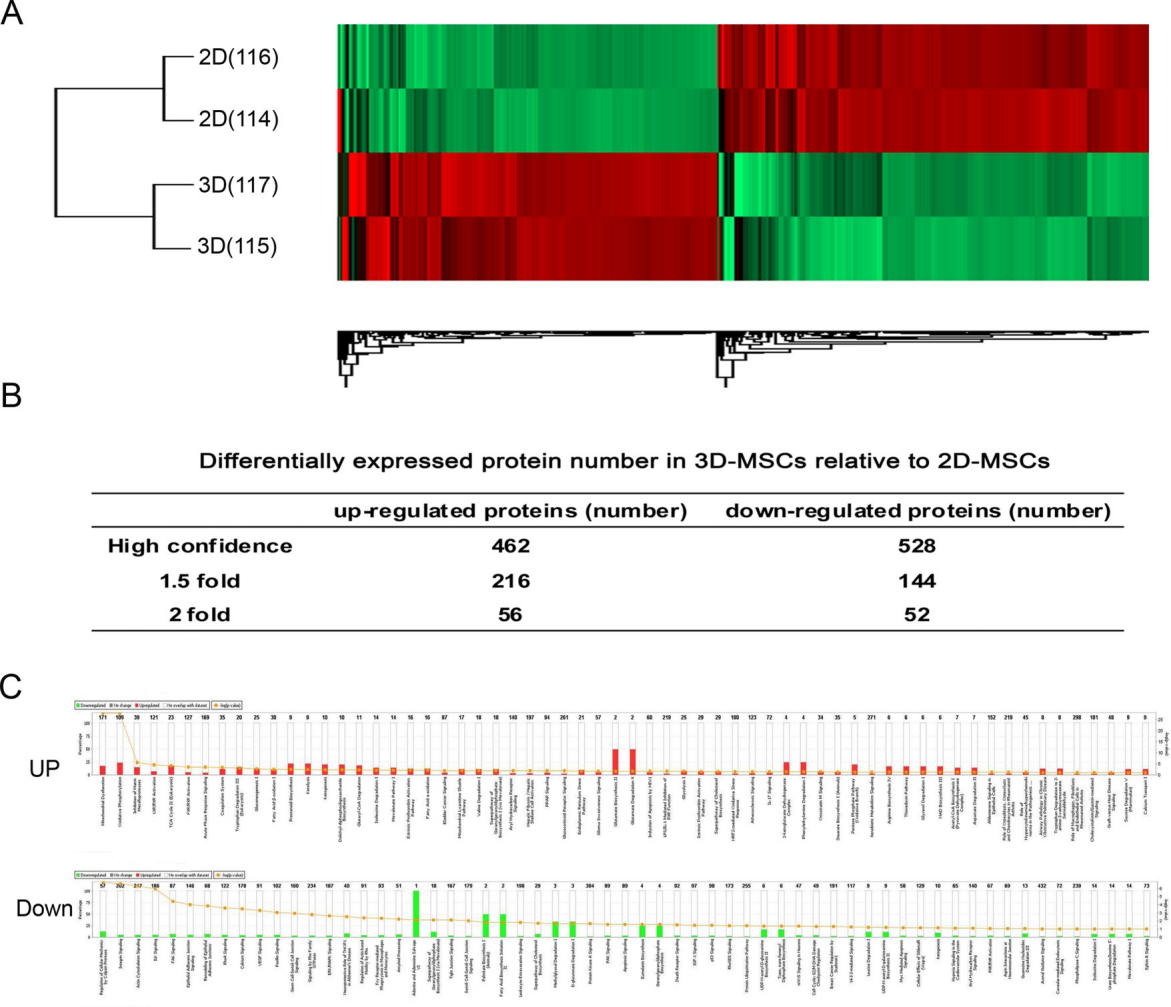
Quantitative proteomics analysis of MSCs cultured on 3D substrates and 2D surfaces. A. Hierarchical clustering of analysis with differentially expressed proteins from 2D-MSCs and 3D-MSCs. B. The differentially expressed proteins of 2D-MSCs and 3D-MSCs. C. Functional categorization of the up-regulated or down-regulated proteins in 3D-MSCs compared to 2D-MSCs. All up-regulated or down-regulated proteins were categorized according to the first-class function annotated by CyanoBase. The bars represent the number of proteins in each functional category.

As expected, we have shown that 2D-MSCs and 3D-MSCs display different biological characteristics, particularly immunomodulatory characteristics. These led us to quantify the differences in protein expression using iTRAQ based quantitative mass spectrometry. The experimental design of the quantitative proteomics and the iTRAQ labeling scheme are diagrammed as Fig 5A. In the experiment, two biological replicates of 2D-MSCs and 3D-collagen MSCs were collected, respectively. The reproducibility of the quantitation was evaluated through correlation of the iTRAQ ratios from two biological replicates (data not shown). In total, 4000 proteins were identified, and 990 high-confidence proteins were identified. Among these, 462 proteins were up-regulated and 528 proteins were down-regulated (Fig 5B). To further ensure that all proteins were truly up-regulated or down-regulated, we excluded all proteins with a fold change less than 1.5, even though they were confidently quantitated as indicated by the p value of the t test. After this exclusion, 216 proteins in total were up-regulated and 144 proteins were down-regulated in 3D-MSCs relative to 2D-MSCs (Fig 5B). The top 20 up-regulated proteins (Table 1), such as PTGS2, IL1RN, and IL1B, were considered to play an important role in the MSC immunosuppressive process [27]. The top 20 down-regulated proteins, such as galectin, were associated with cell-cell interaction and indirectly play an immunomodulatory role [28]. All 360 differentially expressed proteins were functionally categorized according to the first-class functions annotated by CyanoBase (Fig 5C) [29]. The results showed that in the canonical pathway category, 163 terms were enriched in the 3D-up-regulated proteins and 188 terms were enriched in the 3D-down-regulated proteins (Fig 5C). Of them, many pathways were related to cell migration, cell adhesion, cell-cell interaction and immunomodulatory function.

**Table 1:** Top 20 of up-regulated and down-regulated proteins in 3D-MSCs v.s. 2D-MSCs.

Up-regulated proteins		Down-regulated proteins	
Protein N	Name	Protein N	Name
264	PTGS2	386	TAGLN
2420	AHSG	972	MARCKS
3281	Vitronectin	2364	PPP1R14B
2594	APOH	1141	TPM2
1784	AFP	66	CALD1
2088	FGF2	967	FABP3
2055	HBA1	921	HSPB6
660	TFPI2	1263	CNN2
1265	IL1RN	1583	PEA15
523	PTGS1	735	NNMT
1563	GC	545	S100A4
1161	MMP3	122	TPM1
1958	IL1B	677	Galectin-3
1447	SERPINC1	907	CAPNS1
756	A2M	1864	TMSB4X
1144	FTH1	2	AHNAK
1237	FKBP11	1172	S100A6
270	SERPINB2	2040	STMN1
1311	WNT5A	771	ARF4
2099	ITIH3	2712	PHLDB2

Because the protein-protein interaction (PPI) network better reflects the biological status of the cells compared to single proteins, the 216 up-regulated proteins and 144 down-regulated proteins were used to construct PPI networks. In the regulator effect network analysis, we observed top 5 regulator effect networks of disease and function involving the “activation of antigen presenting cells”, “activation of myeloid cells”, “activation of phagocytes”, “recruitment of macrophages”, “release of prostaglandin”, and “inflammatory response” in the 3D-up-regulated proteins, and “cell movement of endothelial cells”, “cell spreading of fibroblast cell lines”, “engulfment of cells”, “growth of tumor”, “migration of tumor cell lines”, “organismal death”, “inflammation of organ”, “immune response of tumor cell lines”, and “proliferation of cells” in the 3D-down-regulated proteins(Tables 2 and 3).

**Table 2:** The regulator effect networks of up-regulated proteins in 3D-MSCs V.S. 2D-MSCs.

ID	Regulators	Disease &Functions	Consistecy Score
1	AGT, APP, CD28, CD44, Cg, CHUK, CSF2, EGF, EGR1 (+24 more)	activation of antigen presenting cell (+14 more)	7979.158
2	AGT, Alpha catenin, CD28, EGF, EGR1, ERK, FGF2 (+18 more)	activation of myeloid cells (+12 more)	74.246
3	AGT, Alpha catenin, APP, CD28, CD3, CD44, EGF (+39 more)	activation of antigen presenting cell (+18 more)	61.988
4	AGT, CD28, CD3, FGF2, FGFR1, FN1, IFNG, IKBKB, IL1 (+19 more)	activation of myeloid cells (13 more)	59.883
5	CD28, EGF, EGR1, FGF2, IFNG, IGF1R, IKBKB,IL1 (+12 more)	activation of myeloid cells (11 more)	33.054

**Table 3:** The regulator effect networks of down-regulated proteins in 3D-MSCs v.s. 2D-MSCs.

ID	Regulators	Disease &Functions	Consistecy Score
1	EGFR, GNA12, IL5, MGEA5, MKL1, MKL2, SMAD4, SRF (+4 more)	cell movement of endothelial cells (+1 more)	11.156
2	ACOX1, AGT, CD38, estrogen receptor, FGF10, SREBF1(+1 more)	engulfment of cells, growth of tumor (+4 more)	9.6
3	ACOX1, APP, ATF4, CD38, CEBPA, IgG, SMAD3	inflammation of organ, polymerization of filaments (+1 more)	5.019
4	EGFR, HRAS, SMAD4	immune response of tumor cell lines	4.025
5	IgG	proliferation of cells	-4.491

### Lymphocyte behavior and immune suppression of MSCs in 3D scaffold culture

Based on the above data of the 3D-MSCs, we intended to confirm whether 3D-MSCs have stronger immunosuppressive effect on immune cells compared with 2D-MSCs to further understand the possible immunomodulatory status of MSCs in vivo.

**Fig 6:**
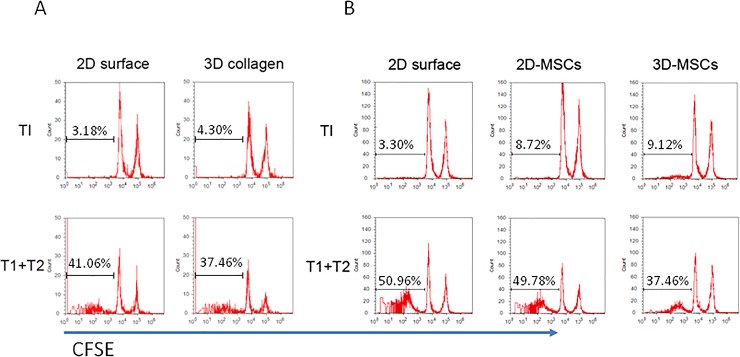
Effects of the 3D culture system on MSC proliferation of T lymphocytes. A. Effects of 3D substrates on the proliferation of T lymphocytes. CFSE-labeled dividing cells after 3 days of culture in 3D substrates and 2D surface are shown. CFSE-labeled T cells from healthy donor used as responders (T1) and un-labeled T cells from another related healthy donor treated with mitomycin C (T2) used as stimulators were cultured in 3D substrates or 2D surface. On day 3, the responder cells were harvested and analyzed by FACS. B. Immune suppressive capacities of MSCs toward T lymphocytes for the 3D culture system and traditional 2D culture system. CFSE labeled responder cells (T1) were stimulated with allogeneic stimulator cells (T2) and co-cultured with 2D-MSCs and 3D-MSCs. After 5 days of co-culture, the cells were harvested and analyzed by FACS.

First, we tested the ability of the 3D scaffolds to stimulate lymphocyte proliferation by MLC. CFSE-labeled T lymphocytes (T1) were used as responders and motimycin C treated T lymphocytes (T2) were used as stimulators, and co-cultured with different 3D substrate. As shown in Fig 6A, cell proliferation was not observed in the presence of 3D substrate after 3 days. This confirmed previous reports on the biocompatibility of collagen, chitosan and PLGA 3D scaffolds and excluded the influence of 3D substrate on immune cells. As shown in Fig 3A, Fig 4 and Fig 5, we postulated that 3D-MSC may have the immune suppressive function. To confirm this, we evaluated the proliferation of T cells labeled with CFSE in co-culture with 2D-MSCs or 3D-MSCs, respectively. As shown in Fig 6B, 2D-MSCs had significant suppressive effects on the proliferation of responded T cells (T1). Furthermore, 3D-MSCs showed stronger suppressive effects on the proliferation of responded T cells (T1) compared to 2D-MSCs. These suppressive effects were also confirmed by the MTS tests on responded T cells after co-culture with 2D-MSCs or 3D-MSCs (data not shown).

## Discussion

Human MSCs are particular interest due to their self-renewal and multipotency properties [3, 30], as well as their immunomodulatory capacities [28, 31], which are attractive for cellular therapies. However, the majority of MSC characteristics that were examined are based on 2D culture system, which was different from the in vivo niches of MSCs. The 3D culture influences the properties of MSCs including cell adhesion, migration, self-renewal, and differentiation, but whether immunomodulatory characteristics were also affected remains unknown. In the present study, we developed three types of 3D culture systems using different substrates to investigate the common immunomodulatory characteristics of human 3D-MSCs.

We firstly observed the growth of human 3D-MSCs. They presented with a less flat morphology and smaller size compared with 2D-MSCs (data not shown). These changes in the morphology of 3D-MSCs indicate that a specific 3D geometry may be responsible for promoting cell multipotency [32] and other biological function. Additionally, the higher proliferation rate and percentages of S phase also suggest that 3D geometry influences the growth characteristics of MSCs.

The stemness of MSCs is the basic characteristic of these cells. The up-regulated expression of Oct4 and Nanog in all 3D-MSCs groups suggest that 3D geometry promotes multipotency maintenance without regard to the differences of substrates. The up-regulated expression of osteogenic-related genes and adipogenic-related genes in all 3D-MSCs groups after osteogenic- and adipogenic-induction indicated that 3D-MSCs maintained a higher differentiation potential compared with 2D-MSCs. The higher multipotency and differentiation potential indicated that 3D culture supports stemness maintenance of MSCs and that a new balance may be established in 3D culture systems.

After the stemness of MSCs was determined, we focused on the influence of 3D culture on the common immune properties of MSCs. Similar MSC marker expression was observed in 3D-MSCs and 2D-MSCs, whereas surprisingly, the expression of surface CD73 is down-regulated in all 3D culture systems. CD73 (Ecto-50-nucleotidase, e-5NT) is a rate-limiting enzyme in the extracellular metabolism of ATP, catalyzing the final step of the conversion of AMP to adenosine. Because this enzyme shifts the balance from pro-inflammatory ATP to anti-inflammatory adenosine, it is considered an important regulator of inflammation and immunity [33]. Based on the function of CD73 and our results, the CD73 expression level must be maintained or up-regulated. Previous studies showed that the activity of CD73 is not only in the cytosolic membrane-bound form but also in the extracellular membrane-bound form, and the soluble 5’-nucleotidase form was also present in cells that retained ecto-5'-nucleotidase activity [34]. Therefore, we hypothesized that the down-regulated surface expression of CD73 was due to the shift of the extracellular membrane-bound form to the cytosolic membrane-bound form or extracellular soluble form. We then further evaluated the immunogenicity of 3D-MSCs, and found that all 3D-MSCs negatively expressed the co-stimulatory molecules, CD80 and CD86, and HLA-DR, suggesting that MSCs retain low immunogenicity properties even under 3D culture systems. The immunophenotypic pattern of 3D-MSCs suggests that 3D-MSCs themselves do not provoke an immune response and may play different roles in the immunomodulatory process.

Together with the changes of immunophenotypic pattern, the common immunomodulatory characteristic of 3D-MSCs was further identified by the global gene and proteome analyses. The genes microarray demonstrated that distinct expression patterns of genes show high similarity among the 3D-MSCs. These were consistent with our previous studies on ES cells [20]. Many of the differentially expressed genes from 2D-MSCs and 3D-MSCs, such as IL1B, IL1RA, IL6ST, TGFBR and TNFSF, correlate with the inflammation and immune regulation of MSCs. For example, IL1B, which may be necessary for the early stage of tissue repair by mediating inflammation, and IL1RA, a natural inhibitor of IL-1 that competes with IL-1 in binding to the IL-1 receptor, are involved together in immune regulation [35]. The differentially expressed genes were commonly enriched in signaling pathways, such as WNT signaling pathway, Toll-like receptor signal pathway, and PPAR signaling pathway. These signaling pathways play important roles in the immune regulation of MSCs. For example, MSCs express a number of TLRs that are activated by TLR ligands leading to the modulation of the differentiation, migration, proliferation, survival, and immunosuppression capacities of MSCs [36].

Because of the great similarity of genes expression in the three types of 3D-MSCs, we selected the 3D-MSCs in collagen substrate culture as the representative 3D-MSCs for quantitative proteomics study. We found that many differentially expressed proteins between 3D-MSCs and 2D-MSCs were associated with immune regulation, particularly immune suppression. Among the up-regulated proteins related to immune regulation, COX2 is in 3D-MSCs. Its biological function is primarily in the synthesis and secretion of prostaglandin E2 (PGE2) which drives the transition of resident macrophages from the classic proinflammatory M1 phenotype toward a more poorly defined M2 phenotype in which the cells secrete anti-inflammatory mediators, such as interleukin-10 (IL-10) and interleukin-1 (IL-1) receptor antagonist [25, 27]. Up-regulation of COX2 in our study indicates that 3D culture may activate the MSC similar to pro-inflammation factors and play an immunosuppressive role by secreting the anti-inflammation factors. The enriched regulatory networks of differentially expressed proteins also suggest the same.

Collectively, the 3D-MSCs played significantly greater immunosuppressive capacities on the proliferation of T lymphocytes compared to 2D-MSCs. This indicates that MSCs might play a stronger immunosuppressive role in vivo, which provides a basis for MSC transplantation in clinical practice.

Although our immunophenotypic and microarray analysis suggest that 3D geometry commonly affects immunomodulatory characteristics of MSCs, and primarily play an immune suppressive role on immune effectors. We still need confirm the immune suppression capacities of 3D-MSCs on immune effector cells by MLC. Previous studies showed that the immunosuppressive ability of MSCs is not constitutive and is induced by the inflammatory microenvironment [37]. In our study, we found that 3D-MSCs presented with stronger immune suppression capacities on T cell proliferation compared with 2D-MSCs, and other recent studies [38] showed the same function on macrophages. Therefore, we speculate that the topological structure changes of MSCs induced by 3D culture influence the immunomodulatory capacities of MSCs on immune effector cells, both in innate immunity and adaptive immunity, by shifting the environment from pro-inflammatory to anti-inflammatory.

In our study, we determine the common change of immunomodulatory characteristics of 3D-MSCs, and confirm the higher immunosuppressive ability of the 3D-MSCs compared with 2D-MSCs. This suggests that the activation of the immunosuppressive function of MSCs in vivo is not only due to inflammatory factors but also to the 3D geometry of the MSCs. The 3D scaffold culture system provides a 3D microenvironment similar in vivo, which is a useful culture system for future stem cell research.

## Conclusions

Three different 3D culture systems were constructed with three different substrates for MSCs. The results demonstrate that all 3D substrates supported MSC growth. The 3D culture commonly affects immunomodulatory characteristics of MSCs, and the changes of characteristics were primarily caused by the alteration in dimension but not the biomaterials. Our findings demonstrate that the 3D geometry of substrate promotes the immune suppression of MSCs, and this could provide a promising system for stem cell-based clinical research.

## Supporting information

S1 Table Primers used in this study. (DOC)

S1 Data MSCs on chitosan V.S. 2D. (XLSX)

S2 Data MSCs on collagen V.S. 2D. (XLSX)

S3 Data MSCs on PLGA V.S. 2D. (XLSX)

S4 Data Result analysis of all data. (XLSX)
