# Supplementary material for: Substrate-independent immunomodulatory characteristics of mesenchymal stem cells in three-dimensional culture
Source: PLoS One. 2018 Nov 8;13(11):e0206811. doi: 10.1371/journal.pone.0206811 (PMC6224081; doi:10.1371/journal.pone.0206811)
Supplement: S1 Table — (DOC) [file pone.0206811.s001.doc]

**S1 Table. Primers used in this stud**y

| S.N | Gene | Sequence 5'--3' |
| --- | --- | --- |
| 1 | Oct-4-F | GCAGCTTGGAAGGCAGAT |
|  | Oct-4-R | TGGATTTTAAAAGGCAGAAGACTTG |
| 2 | Nanog-F | ACCTATGCCTGTGATTTGTGGG |
|  | Nanog-R | GACCTTGTCTTCCTTTTTTGCGA |
| 3 | Sox2-F | GCCGAGTGGAAACTTTTGTCG |
|  | Sox2-R | GCAGCGTGTACTTATCCTTCTT |
| 4 | RUNX2-F | TGTCATGGCGGGTAACGAT |
|  | RUNX2-R | AAGACGGTTATGGTCAAGGTGAA |
| 5 | BMPR2-F | CCACCTCCTGACACAACACC |
|  | BMPR2-R | GACCTTGTTTACGGTCTCCTG |
| 6 | OPN-F | ACTCGAACGACTCTGATGATGT |
|  | OPN-R | GTCAGGTCTGCGAAACTTCTTA |
| 7 | Cfd-F | GGTCACCCAAGCAACAAAGT |
|  | Cfd-R | CCTCCTGCGTTCAAG-TCATC |
| 8 | PPARγ-F | CCTATTGACCCAGAAAGCGATT |
|  | PPARγ-R | CATTACGGAGAGATCCACGGA |
| 9 | C/EBPα-F | AGGAACACGAAGCACGATCAG |
|  | C/EBPα-R | CGCACATTCATTGCACAA |
| 10 | IL1A-F | CATAGCCAGGAAACTCTGC |
|  | IL1A-R | TTGAATGAAACAAGAATGCC |
| 11 | IL1B-F | ATGATGGCTTATTACAGTGGCAA |
|  | IL1B-R | GTCGGAGATTCGTAGCTGGA |
| 12 | IL1RN-F | TACCTGCCAAGAGCGAGG |
|  | IL1RN-R | GGGGGTTCTTTCTTCCTCTG |
| 13 | IL6ST-F | TCAGTCCTGAATCTCCAGTTGT |
|  | IL6ST-R | CTGTTCAAGCTGTCCGAATGTA |
| 14 | HGF-F | ACGAACACAGCTATCGGGGTA |
|  | HGF-R | CATCAAAGCCCTTGTCGGGAT |
| 15 | EGF-F | TGCAACTGTGTTGTTGGCTACATC |
|  | EGF-R | TGGTTGACCCCCATTCTTGAG |
| 16 | GAPDH-F | GGTCACCAGGGCTGCTTTTA |
|  | GAPDH-R | GAGGGATCTCGCTCCTGGA |
